# aSynPEP-DB: a database of biogenic peptides for inhibiting α-synuclein aggregation

**DOI:** 10.1093/database/baad084

**Published:** 2023-11-27

**Authors:** Carlos Pintado-Grima, Oriol Bárcenas, Valentín Iglesias, Jaime Santos, Zoe Manglano-Artuñedo, Irantzu Pallarès, Michał Burdukiewicz, Salvador Ventura

**Affiliations:** Institut de Biotecnologia i de Biomedicina and Departament de Bioquímica i Biologia Molecular, Universitat Autònoma de Barcelona, Bellaterra, Barcelona 08193, Spain; Institut de Biotecnologia i de Biomedicina and Departament de Bioquímica i Biologia Molecular, Universitat Autònoma de Barcelona, Bellaterra, Barcelona 08193, Spain; Institut de Biotecnologia i de Biomedicina and Departament de Bioquímica i Biologia Molecular, Universitat Autònoma de Barcelona, Bellaterra, Barcelona 08193, Spain; Institut de Biotecnologia i de Biomedicina and Departament de Bioquímica i Biologia Molecular, Universitat Autònoma de Barcelona, Bellaterra, Barcelona 08193, Spain; Center for Molecular Biology of Heidelberg University (ZMBH), Heidelberg 69120, Germany; Institut de Biotecnologia i de Biomedicina and Departament de Bioquímica i Biologia Molecular, Universitat Autònoma de Barcelona, Bellaterra, Barcelona 08193, Spain; Institut de Biotecnologia i de Biomedicina and Departament de Bioquímica i Biologia Molecular, Universitat Autònoma de Barcelona, Bellaterra, Barcelona 08193, Spain; Institut de Biotecnologia i de Biomedicina and Departament de Bioquímica i Biologia Molecular, Universitat Autònoma de Barcelona, Bellaterra, Barcelona 08193, Spain; Clinical Research Centre, Medical University of Białystok, Kilińskiego 1, Białystok 15-369, Poland; Institut de Biotecnologia i de Biomedicina and Departament de Bioquímica i Biologia Molecular, Universitat Autònoma de Barcelona, Bellaterra, Barcelona 08193, Spain

## Abstract

Parkinson’s disease (PD) is the second most prevalent neurodegenerative disorder, yet effective treatments able to stop or delay disease progression remain elusive. The aggregation of a presynaptic protein, α-synuclein (aSyn), is the primary neurological hallmark of PD and, thus, a promising target for therapeutic intervention. However, the lack of consensus on the molecular properties required to specifically bind the toxic species formed during aSyn aggregation has hindered the development of therapeutic molecules. Recently, we defined and experimentally validated a peptide architecture that demonstrated high affinity and selectivity in binding to aSyn toxic oligomers and fibrils, effectively preventing aSyn pathogenic aggregation. Human peptides with such properties may have neuroprotective activities and hold a huge therapeutic interest. Driven by this idea, here, we developed a discriminative algorithm for the screening of human endogenous neuropeptides, antimicrobial peptides and diet-derived bioactive peptides with the potential to inhibit aSyn aggregation. We identified over 100 unique biogenic peptide candidates and ensembled a comprehensive database (aSynPEP-DB) that collects their physicochemical features, source datasets and additional therapeutic-relevant information, including their sites of expression and associated pathways. Besides, we provide access to the discriminative algorithm to extend its application to the screening of artificial peptides or new peptide datasets. aSynPEP-DB is a unique repository of peptides with the potential to modulate aSyn aggregation, serving as a platform for the identification of previously unexplored therapeutic agents.

**Database URL:**  https://asynpepdb.ppmclab.com/

## Introduction

The progress in medical sciences has remarkably increased human life expectancy worldwide. However, aged populations are vulnerable to different risks, including an increased likelihood of developing neurodegenerative disorders (1). Parkinson’s disease (PD) is the second most prevalent neurodegenerative disease and affects 1% of the population over 60 years (2). Since current treatments are purely symptomatic and become ineffective at later stages of the disease (3), there is an urgent need to devise new disease-modifying therapies that can slow down or halt the progression of PD and related conditions.

The aggregation of a small protein, α-synuclein (aSyn), into amyloid fibrils in the brain’s dopaminergic neurons is the primary hallmark of PD (4, 5). For this reason, many efforts have focused on identifying drugs able to specifically target aSyn and prevent its aggregation (6–10). However, the intrinsically disordered nature of aSyn complicates the rational design and development of new therapeutic entities that can selectively target aSyn toxic species. Recently, we demonstrated that a particular subset of amphipathic and cationic α-helical peptides binds aSyn toxic oligomers and fibrils with nanomolar affinity, arresting pathogenic aggregation (11). We initially identified this effect for the bacterial antimicrobial PSMα3 peptide, and subsequentially used peptide redesign to delineate the key physicochemical properties responsible for this activity. Our quest for a human peptide exhibiting analogous physicochemical characteristics led us to identify LL-37. We demonstrated that this peptide binds tightly to aSyn aggregates, blocking amyloid progression and mitigating aSyn oligomers associated cellular damage. Remarkably, LL-37 is constitutively expressed in the brain and gut, two tissues where aSyn aggregation occurs in disease (12), thus suggesting a role in controlling aSyn aggregation in disease-relevant tissues. This discovery paved the way to explore human endogenous peptides possessing similar properties, with the aim to expand the repertoire of potential molecules to combat PD (13).

The identification of LL-37 suggested that other biogenic peptides may similarly exert chaperoning activities against aSyn aggregation. In this work, we harnessed the knowledge gained from our empirically derived data to search for undiscovered aSyn-targeting peptides that could be present in PD-relevant tissues, such as the brain or gastrointestinal tract. With that aim, we computationally screened three datasets of peptides encompassing (i) human endogenous neuropeptides and antimicrobial peptides (AMPs), (ii) human endogenous gut-microbiome peptides and (iii) bioactive peptides that might be incorporated from diet and absorbed by intestinal cells (14–16). Considering the pivotal role of the gut-brain axis and dietary patterns in the onset and progression of PD (17), we perceive the latter dataset as a particularly promising source of bioactive molecules.

We identified over 100 peptides predicted to inhibit the aggregation of aSyn by targeting toxic species, which were collected in a repository named aSynPEP-DB. The database displays the key features relevant for inhibition and a structural model of the peptide, as well as additional relevant information, such as tissue expression, associated pathways or membrane permeability, among others. Alongside, aSynPEP-DB provides access to the discriminative algorithm employed for screening, allowing users to extend the analysis to other peptide datasets or even design new artificial peptides. We believe these features make the aSynPEP-DB a unique resource for assisting the development of peptide-based therapeutic strategies for PD and related synucleinopathies.

## Methods

### Dataset generation

**Figure 1.**
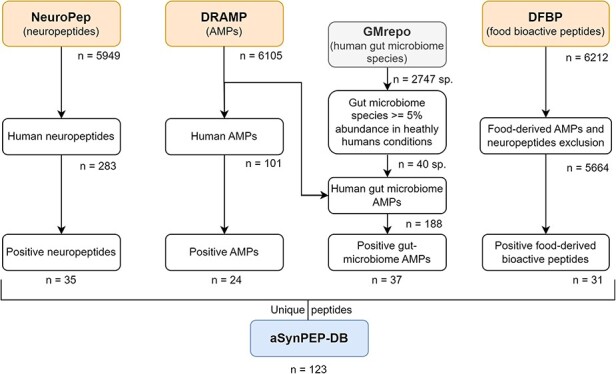
Data curation scheme. Peptides collected in the aSynPEP-DB are obtained from independent source datasets of biogenic peptides. These include human neuropeptides (NeuroPep database), human and human gut microbiome AMPs (DRAMP database) and food-derived bioactive peptides (DFBP database). To select which bacterial species were found in the gut microbiome, the GMrepo database was employed. A total of 123 unique peptides were obtained with the discriminative algorithm.

The aSynPEP-DB collects human endogenous peptides that could be present in PD-relevant tissues, such as the brain or gastrointestinal tract, in addition to naturally obtained food-derived bioactive peptides. The experimentally validated peptides found in our previous work demonstrated that AMPs might have anti-amyloid potential (18). For this reason, dedicated peptide sources of manually curated neuropeptides and AMPs⁠ -NeuroPep (19) and DRAMP (20), respectively, were selected for the initial screening (Figure 1). A total of 283 human neuropeptides and 101 human AMPs were retrieved for further analysis. In addition, given the increasing evidence of the associations between human microbiota and PD, AMPs produced by human gut bacteria were also considered. To obtain a list of bacteria species found in the human gut microbiome, the manually curated database GMrepo (21) was employed. A total of 40 gut microbiome species were obtained with an abundance ≥5% in healthy humans (Supplementary Table S.1). DRAMP listed 188 different AMPs for these species. Finally, food-derived bioactive peptides were obtained from DFBP (22). AMP and neuropeptide classifications were excluded since they were already considered as human endogenous peptides by NeuroPep and DRAMP. Only peptides composed of natural amino acids and without modifications were considered for the next screening step.

### Discriminative algorithm for screening peptide candidates

aSyn toxic oligomers and fibrils expose hydrophobic clusters to the solvent while retaining a highly anionic character (23, 24). These two properties can be targeted by a complementary hydrophobic and cationic molecule, a notion that inspired the design and identification of amphipathic and cationic helical peptides capable of selective targeting aSyn toxic species without recognizing functional monomeric aSyn (11). This research experimentally demonstrated that, regardless of the specific sequence, α-helical peptides are able to bind aSyn toxic species as long as they retain their hydrophobic and cationic character in opposite faces of the α-helix. Thus, an adequate formulation of these physicochemical properties is enough to identify previously unreported active human peptides, as previously demonstrated for LL-37.

With this knowledge, we developed a heuristic discriminative algorithm to screen human endogenous peptides obtained from NeuroPep, DRAMP and DFBP with the potential to inhibit aSyn aggregation. Final positive hits are peptides that meet the following criteria:

α-helical propensity: the propensity of peptides to form α-helical secondary structure was assessed with AGADIR (25) in default configuration (no N- or C-terminal protection, pH 7, temperature 278 K and ionic strength 0.1). The tool provides numerical outputs with the α-helical propensity based on experimental peptidic data. Aside from the reliability of the algorithm, providing a numerical output was more convenient for establishing informative decision thresholds than visual representations of the peptides’ secondary structure. Peptides with an AGADIR score ≥1 were considered to have a sufficient α-helical tendency.

Length: the peptide length intervals were selected based on the assumption that peptides exhibit an α-helical propensity. The minimum peptide length was established at 18 residues which consist of 5 helical turns whereas the maximum peptide length was set to 54, corresponding to 15 helical turns.

Amphipathicity: the two-face distribution in the helical peptide of hydrophobic and polar residues was computed based on the hydrophobic dipole moment (uH) obtained from an array of hydrophobicity values (26). Peptides with uH ≥0.2 were considered confident amphipathic entities.

Positive net charge per residue (NCPR): NCPR was assessed by averaging the global net charge per residue obtained using the Henderson-Hasselbalch equation at pH 7. Only peptides with at least one positive charged residue per 18 residues were considered.

Peptides meeting these criteria were considered positive hits. However, it is also possible that the full-length peptide is negative but its sequence contains a shorter region with putative inhibitory properties. Therefore, when full-length peptides are deemed negative, a sliding window of 18 residues is run along the sequence searching for shorter inhibitory regions. If found, the longest positive stretch is reported. After the screening with the discriminative algorithm, a total of 123 unique peptides were obtained from the original datasets (Supplementary Figure S.1).

However, in addition to biogenic peptides, users might be interested in finding additional peptides from external datasets not considered, given the aim of this work. For this reason, we have embedded the discriminative algorithm in the database so that users can run their own predictions independently, which can be accessed under the ‘Algorithm’ tab of the database (please refer to the Supplementary Figure S.2a for the flowchart and Supplementary Figure S.2b for a screenshot of the website). The discriminative algorithm allows the introduction of synthetic peptides and/or additional source datasets to screen possible inhibitory peptides. Given the possibility of α-helical conformations being adopted according to the environmental factors or upon binding to a partner, the algorithm leaves the AGADIR helical threshold as an input variable: by reducing it, disordered peptides that can potentially become structured upon binding can be included. Besides, peptide ends can be acetylated or amidated for N- or C-terminus protection, respectively, to mimic cellular conditions, as in the original AGADIR software (25). The tool calculates the physicochemical properties required for inhibition and displays the results in a tabular format along with the final decision for each peptide. Helical wheels are generated for peptides meeting the AGADIR threshold and can be accessed by clicking on the peptide ID.

### Additional predicted features

In addition to the physicochemical properties required for classification, the database is enriched with features that extend structural information and possible therapeutic uses. Peptide property predictors were selected according to the reproducibility and software availability of dedicated tools (27).

AlphaFold peptide structure: given the success of AlphaFold in accurately predicting protein structures from sequences (28), predicted peptide structures were obtained from ColabFold (29) to provide practical 3D representation and support the AGADIR helical propensity.

pH-dependent order/disorder transitions: polypeptide sequences can adopt conformational transitions from disordered to ordered structures upon fluctuations in pH (30). Such transitions could disrupt the peptide’s helical conformation and therefore its inhibitory capacity. Also, the pH-dependent conformational information may be critical for non-therapeutic applications in the specific identification of aSyn toxic species, including diagnostics or probe design. aSynPEP-DB incorporates DispHred predictions (31, 32) to account for this possibility. DispHred pH-dependent disorder predictions were run between pH 3.0 and pH 9.0 (step size of 0.1).

Cytotoxicity: peptides listed in the aSynPEP-DB might be potentially used as new molecular entities to treat PD, where cytotoxicity becomes a crucial property to be considered. The ToxIBTL (33) predictor of peptide toxicity is employed to report the expected cytotoxic effect.

Blood-brain barrier (BBB) permeability: the ability to cross the BBB is one of the major bottlenecks of therapeutic drugs (34). This is of tremendous importance since PD pathogenicity mainly occurs in the central nervous system. In agreement, BBB predictions from BBPpred (35) have been incorporated into peptide records.

Cell penetration: apart from endothelial cells in the BBB, other relevant cell types such as those located in the gut might play an important role in the pharmacokinetics of these molecules. Therefore, each record also collects predictions from the cell-penetrating peptide tool BchemRF-CPPred (36).

### Database implementation

The aSynPEP-DB was implemented in the form of a user-friendly database that includes all listed candidates in a tabular format. Moreover, each individual entry is populated with more in-depth information on the characteristics of each peptide. The database was built upon the DataTables plug-in for the jQuery Javascript library with custom CSS and JS. The website framework was deployed using the Quarto framework. Peptides were linked to their source repositories and precursor proteins. When the source protein was not available, BLAST searches were employed to screen the most confident match. For gut-microbiome derived peptides, the source organism was retrieved from GMrepo and linked to their corresponding NCBI Taxon IDs. Also, existing PubMed IDs collected in the source databases were linked to the record information.

The discriminative algorithm included in the database was written in Python 3.7. The front-end is a combination of HTML, CSS and JS. The server is built using the Django 3.0 web framework and allows for the computation of around 3500 peptides of an average length of 30 residues in a single job.

## Results

### aSynPEP-DB

**Figure 2.**
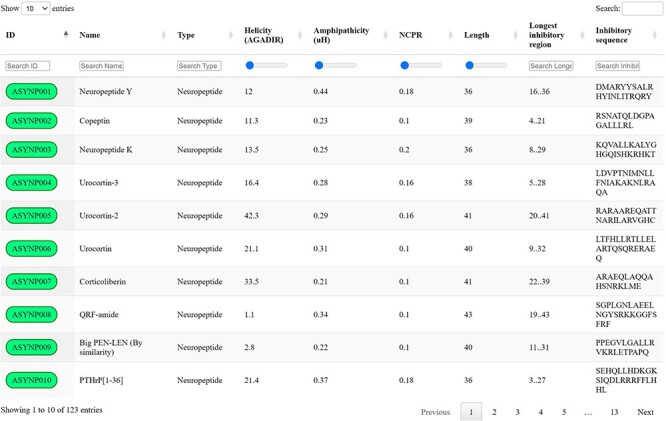
aSynPEP-DB database view. Positive entries are collected in an interactive data table under the ‘Database’ tab of the website. Users can perform general searches using the top right text box or by typing under each specific column. For numerical variables, range sliders are provided to filter entries. All columns can be sorted by clicking on the specific header. The number of displayed entries can be modified with the top left selector. Extended peptide details can be accessed by clicking on each peptide identifier.

Screening the selected datasets with the proposed discriminative algorithm and thresholds led to the identification of 123 unique biogenic peptides with the potential to inhibit aSyn aggregation by targeting toxic species, which were collected in the aSynPEP-DB (https://asynpepdb.ppmclab.com/) (Figure 2). Users can browse the database by typing the peptide ID, name, peptide source or sequence. The search can be easily tailored through the use of range sliders for specific physicochemical features. Multiple filters can be concurrently applied for a more refined search. Besides, the table can be sorted by column simply by clicking on the specific headers. Upon accession to a specific peptide record, users will find a detailed overview of the peptide’s characteristics: aSynPEP physicochemical properties, predicted features, associated expression and related biological pathways. Full database information and code can be found in the website’s ‘Download’ section.

### Case studies

The peptides collected in aSynPEP-DB constitute a source of potential novel molecules for therapeutic development and/or physiological elements potentially connected to PD. To illustrate the key information captured in aSynPEP-DB and its possible translational implications, we have selected a representative candidate of each source dataset (a human neuropeptide, a food-derived bioactive peptide, a human AMP and a gut-microbiome peptide) to illustrate a hypothetical exercise of peptide discovery leveraging this database.

#### A human endogenous neuropeptide: the neuropeptide Y

**Figure 3.**
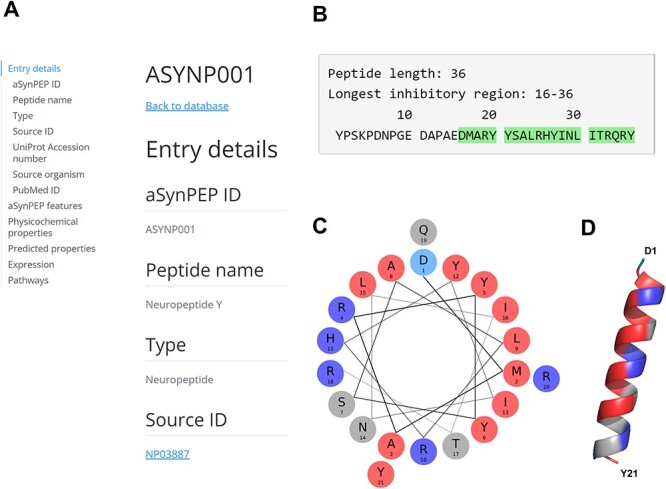
aSynPEP-DB record view for the human neuropeptide Y (ASYNP001). (A) Record view panel showing the information provided for each peptide, including entry details, aSynPEP features, physicochemical and predicted properties, expression and pathway information. (B) Peptide sequence with the inhibitory region detected by the discriminative algorithm highlighted in green. (C) Helical wheel representation for the NPY. Hydrophobic and positively charged residues are colored red and dark blue, respectively. (D) Predicted α-helical conformation obtained for the NPY. The structural model was obtained using AlphaFold through ColabFold. The amphipathic character of the helix is visualized with a hydrophobic face (in red) in front of a second cationic face (in dark blue).

The neuropeptide Y (NPY; ID = ASYNP001) is a 36-residue peptide derived from the precursor proneuropeptide-Y protein and is found across the human brain (37). Full peptide record is displayed upon clicking its identifier (Figure 3A). The aSynPEP discriminative algorithm identifies a short inhibitory region of 21 residues (16-DMARYYSALRHYINLITRQRY-36) at the C-terminus end of the full-length peptide (Figure 3B). This region is highly amphipathic when projected in an α-helical representation (uH = 0.44), having well-defined hydrophobic and cationic faces (Figure 3C). This distribution in two faces with diverging physicochemical character agrees with its predicted α-helical propensity (AGADIR score = 12) and the AlphaFold structural model (Figure 3D).

Consistent with our predictions, NPY has been described to exert potent neuroprotective effects against PD via multiple pathways (38). Additionally, decreased levels of NPY have been documented in the cerebrospinal fluid of PD patients (39). While the influence of NPY on aSyn aggregation is yet to be clarified, the peptide possesses all the essential physicochemical attributes to effectively bind to aSyn’s toxic forms. In addition, NPY is predicted to be non-toxic and cell-penetrating. This information supports a re-examination of the role of this peptide in PD and the mechanisms behind its demonstrated therapeutic activity. Moreover, NPY expression is sensitive to diet composition with variable effects depending on carbohydrate and fat intake (40), suggesting the possibility to upregulate its expression through dietary adjustments.

#### The food-derived bioactive BMAP-28 peptide

**Figure 4.**
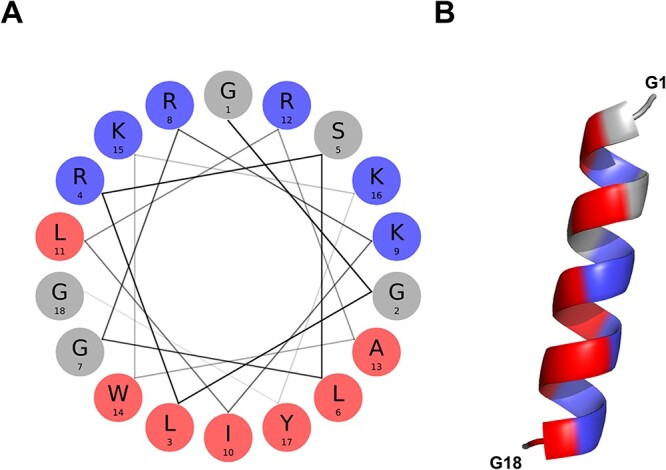
α-helical conformation of the food-derived bioactive BMAP-28 peptide (ASYNP110). (A) Helical wheel visualization of BMAP-28, which holds the highest amphipathicity of all peptides collected in the database (hydrophobic and cationic residues in red and dark blue, respectively). (B) Predicted α-helical conformation obtained for BMAP-28 (AlphaFold).

The BMAP-28 (ID = ASYNP110) is an 18-residue peptide derived from the precursor Cathelicidin-5 bovine protein (41). The full-length peptide (1-GGLRSLGRKILRAWKKYG-18) is identified as a positive hit by the discriminative algorithm, indicating it possesses the attributes necessary for aSyn inhibition. Of note, it is the peptide with the highest amphipathicity of the aSynPEP-DB (uH = 0.64) with a clear cationic face (NCPR = + 0.34) enriched in lysines and asparagines (Figure 4A). The α-helical propensity is predicted by an AGADIR score = 1.6 and supported by the AlphaFold-generated structural model (Figure 4B).

Cathelicidins are a family of AMPs in humans and other mammals with a broad spectrum of antimicrobial activity against bacteria, enveloped viruses and fungi (42). Since the endogenous human cathelicidin-derived peptide LL-37 has been already proved to inhibit aSyn aggregation (11), related biogenic peptides such as BMAP-28 are anticipated to exhibit similar activity. The proposed mechanism of action of BMAP-28 involves, first, its positive net charge, allowing it to penetrate the dense and highly negative fuzzy coat that surrounds aSyn oligomers and fibrils. Then, the hydrophobic face would look for and block inner aSyn hydrophobic residues, which are typically implicated in aggregation and neurotoxicity (43). The strong binding of this kind of peptides stems from their avidity. Once they breach the negative cloud, it is difficult that they would return back to the solution, and they are compelled to persistently engage in interactions with inner hydrophobic surfaces within oligomers and fibrils.

Remarkably, BMAP-28 has been detected in exosomes in the whey of skimmed milk obtained from healthy cows (44). This is because milk contains viable immune cells, which secrete immune-related components, including cathelicidins (45). BMAP-28 is a biogenic short peptide; it is predicted to be non-toxic, cell-penetrating and BBB permeable. This positions it as a food-derived candidate whose therapeutic potential is worth to be assessed.

#### The human antimicrobial lactoferricin-H peptide

Lactotransferrin is an iron-binding protein found in exocrine fluids (46) whose cleavage generates different peptidic chains, including lactoferricin-H (LfcinH; ID = ASYNP074). LfcinH is a 48-residue AMP with a predicted inhibitory region (QWCAVSQPEATKCFQWQRNMRKVRGPPVSCIKRDSPIQ) which encompasses amino acids 7–44. AGADIR predictions (score = 1) and AlphaFold structural model agree that the positive region is structured in α-helical conformation. It also has an amphipathic character (uH = 0.32) and is positively charged at neutral pH (NCPR = + 0.21), becoming a putative candidate to inhibit aSyn aggregation. Interestingly, lactotransferrin proteins have been found to ameliorate dopaminergic neurodegeneration and motor deficits in a mouse model of PD via several mechanisms (47). Whether lactoferrins’ neuroprotective effects are solely associated with the regulation of iron metabolism or they can also directly affect aSyn biology is still unclear, but the presence of LfcinH in aSynPEP-DB suggests that this could be the case.

#### The gut-microbiome peptide OR-7

The Bacteriocin OR-7 peptide (ID = ASYNP044) is an AMP derived from the gram-positive bacteria *Lactobacillus salivarius*, present in the human gut of healthy individuals. It is a 54-residue peptide with a potential inhibitory region of length 20 amino acids at the C-terminal end (34-MGRLQDILLGWATGAFGKTF-54). It corresponds to an amphipathic peptide (uH = 0.32) projected into an α-helical conformation according to both AGADIR and AlphaFold predictions. Given its properties, the presence of this peptide in the gut could have a preventive effect in PD pathogenesis. Growing preclinical evidence shows that gut aSyn can be transported to the brain via the vagus nerve (48), and the interaction of aSyn by bacterial components (49) can modulate its aggregation (11). For these reasons, there is an increasing interest in the usage of microbial-related therapies to safely alleviate PD symptoms (50). Interestingly, the OR-7 peptide is in preclinical stage for its therapeutic effect against other bacteria, preventing gastrointestinal tract infections *in vivo* (51). It might be possible that the same physicochemical properties required for such a bactericidal effect could also play a role in chaperoning aSyn in the human gut (18). If this is the case, gut-microbiome species expressing such peptides could be potentially used as therapeutic probiotics.

## Discussion

In our previous work, we elucidated the physicochemical properties that allow a specific group of peptides to inhibit aSyn aggregation by selectively targeting toxic oligomers and fibrils (11). This paved the way for investigating a vast array of uncharted peptides that may possess similar inhibitory effects against aSyn. In this context, aSynPEP-DB was developed to collect peptides that can be naturally found in disease-relevant tissues in humans. These peptides are candidates for their use as safe therapeutic molecules. In fact, peptides that decrease aSyn spreading has been already proposed in therapeutic intervention in PD (52).

Interestingly, the expression of human endogenous peptides can be upregulated by a variety of substances which can be easily obtained from food (40, 53). This suggests that beyond the direct inhibitory action of food-derived bioactive peptides, other natural compounds could be used to synergistically enhance the expression levels of peptides cataloged in the aSynPEP-DB. For instance, substances such as vitamin D or butyrate upregulate the expression of the AMP LL-37 (53). These same molecules have been linked to the alleviation of PD symptoms (54). As an alternative, recent advances in peptide delivery strategies could be possibly exploited for brain/gut delivery of these potentially therapeutic peptides, including intranasal administration (55, 56) or bioinspired innovative strategies (57). In the case of endogenous gut-microbiome peptides, the usage of probiotics (50), fecal microbiota transplantation (58) or certain dietary choices could be explored as methods alternative to their direct administration.

Some peptides collected in the database may play an evolutionary selected role in modulating aSyn aggregation. Others may serve additional functions, but since they share the required physicochemical properties (amphipathicity, helicity and positive net charge), they can be potentially exploited to fight PD. In this sense, it has been proposed to exist a functional and structural cross-talk between the anti-amyloid, antimicrobial and anti-biofilm activities of certain peptides and small proteins (18). By unraveling human molecules that interact with aSyn aggregates, aSynPEP-DB may define not only novel therapeutic candidates but also new biomarkers to monitor in PD patients.

Overall, the aSynPEP-DB encompasses a limited, yet unique, set of manually annotated peptides curated after a careful dataset selection. We were restrictive both in the selection of the datasets and the physicochemical properties parameters to render a narrow but reasonably confident group of relevant peptides. We are aware that PD-protective bioactive peptides exist outside of the human spectrum (59–61), while others have been synthetically engineered or modified (62, 63). To accommodate these cases, we provide a discriminative algorithm to broaden the search for possible candidates. For each peptide sequence, the tool calculates α-helical propensity, amphipathicity and NCPR. If the peptide (or a region of it) meets the threshold set for each variable, it is considered a positive hit. To our knowledge, this is the first computational tool specifically designed for detecting prospective peptide inhibitors of aSyn aggregation.

The 123 peptides catalogued in the database are naturally found in humans as neuropeptides, AMPs, gut- or food-derived, and collectively possess the physicochemical attributes for inhibiting pathogenic aSyn aggregation. It is important to recall that peptides listed in the aSynPEP-DB are, in its majority, still untested for their desired anti-aggregative features. Therefore, experimental validation remains essential to ascertain the inhibitory efficacy of these molecules, but our preliminary *in vitro* aggregation kinetic experiments strongly suggest that, in many instances, this would be the case (data available upon request). In addition, a notable proportion of the peptides (∼15%, 18/123) have prior associations with PD, as evidenced by comprehensive manual curation of the available literature (Supplementary Table S.2).

Overall, the database and discriminative algorithm we provide in this study opens a previously uncharted molecular landscape to fish novel entities of natural or synthetic origin targeting what is believed to be the pivotal step in the genesis of synucleinopathies: the formation and build-up of toxic aSyn aggregated forms. We anticipate this repository will attract significant interest from the scientific and medical community, given the potential of these peptides, or their derivatives, to modify the course of these debilitating neurodegenerative diseases.

## Supplementary Material

baad084_Supp

## Data Availability

Full record information collected in the aSynPEP-DB can be downloaded from https://asynpepdb.ppmclab.com/download.html. All files needed for deploying the database locally can be found at https://github.com/cpintado7/asynpepdb.
