# Supplementary material for: aSynPEP-DB: a database of biogenic peptides for inhibiting α-synuclein aggregation
Source: Database (Oxford). 2023 Nov 27;2023:baad084. doi: 10.1093/database/baad084 (PMC10681447; doi:10.1093/database/baad084)
Supplement: baad084_Supp [file baad084_supp.zip › suppl_data/aSynPepDB_supplementary-final.pdf]

Supplementary material for

aSynPEP-DB: a database of biogenic peptides for inhibiting a-synuclein aggregation

Carlos Pintado-Grima, Oriol Bárcenas, Valentín Iglesias, Jaime Santos, Zoe Manglano-Artuñedo, Irantzu Pallarès, Michał Burdukiewicz* and Salvador Ventura*

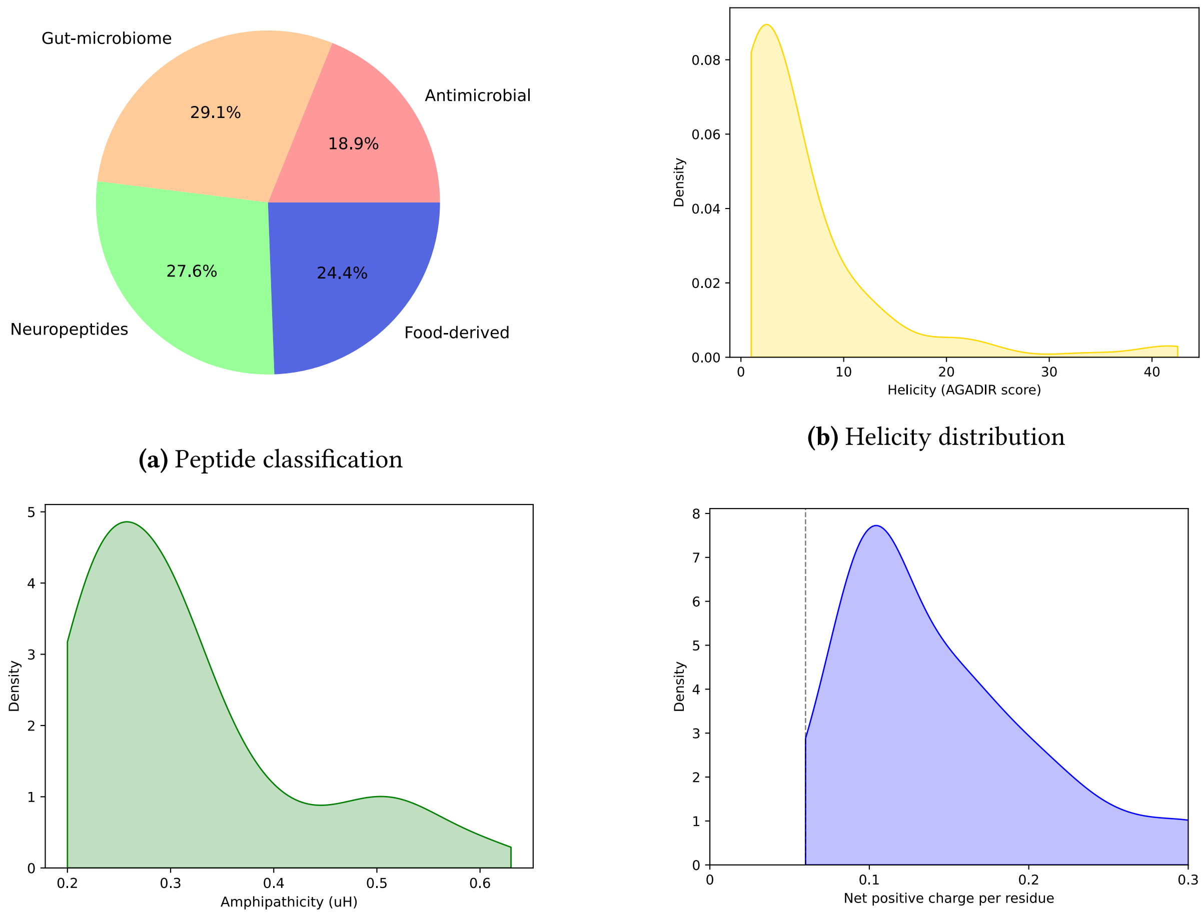

(a) Peptide classification

(b) Helicity distribution

(c) Amphipathicity distribution

(d) NCPR distribution

Figure S.1: aSynPEP-DB statistics displayed in the database, which includes peptide classification and the physicochemical features (helicity, amphipathicity and NCPR) distribution of positive peptides.

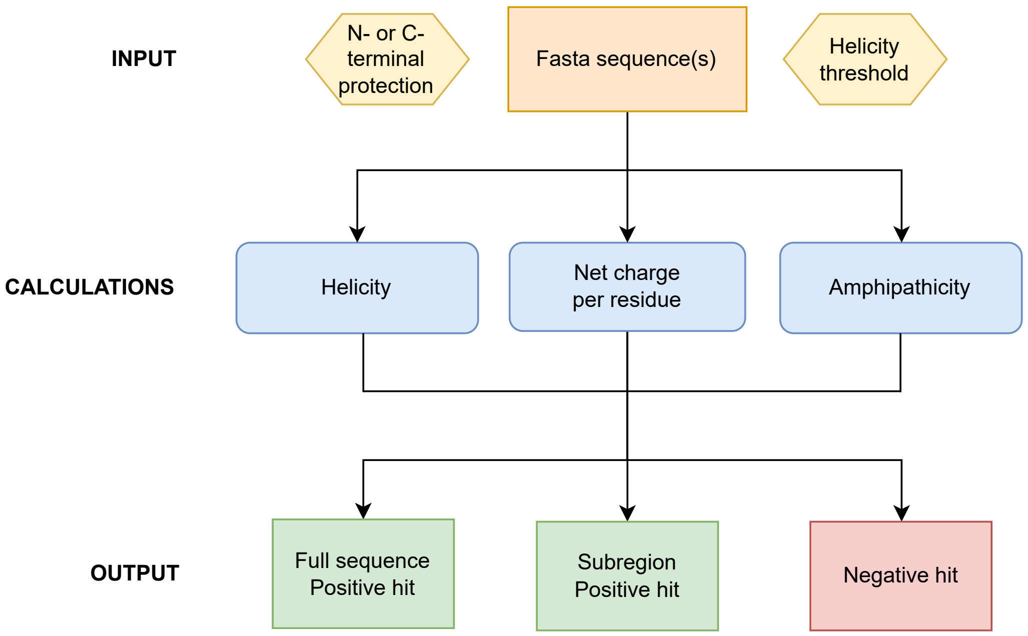

(a) Diagram of the methodology employed in the discriminative algorithm

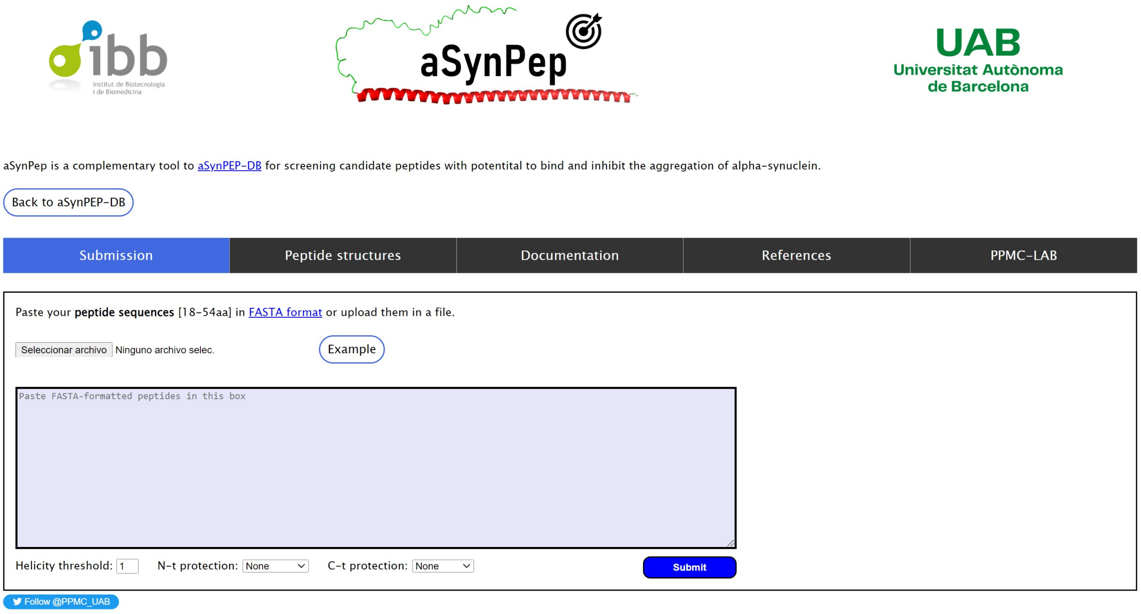

(b) Screenshot of the discriminative algorithm web service

Figure S.2: Discriminative algorithm for the prediction of synthetic peptides or additional datasets. The aSynPEP discriminative algorithm can be accessed from the Algorithm tab of the website. Users can either upload a FASTA file or paste the sequences in the textbox. The AGADIR helical threshold can be adjusted (default is set to 1, as used in the database), and peptide protections in the N- and C- terminus end can be applied.

Table S.1: List of the 40 gut microbiome species with an abundance $\geq$ 5% from healthy humans obtained from GMrepo.

Staphylococcus epidermidis	*Ruminococcus gnavus*	*Clavibacter michiganensis*	*Staphylococcus aureus*
Enterococcus faecium	*Lactobacillus salivarius*	*Lactobacillus plantarum*	*Leuconostoc mesenteroides*
Leuconostoc pseudomesenteroides	*Escherichia coli*	*Klebsiella pneumoniae*	*Lactobacillus animalis*
Bacillus thuringiensis	*Butyrivibrio fibrisolvens*	*Lactococcus lactis*	*Bacillus licheniformis*
Streptococcus salivarius	*Streptococcus mutans*	*Paenibacillus polymyxa*	*Streptococcus uberis*
Enterococcus hirae	*Leuconostoc gelidum*	*Pediococcus acidilactici*	*Lactobacillus sake*
Bifidobacterium bifidum	*Lactobacillus acidophilus*	*Lactobacillus johnsonii*	*Lactobacillus gasseri*
Lactobacillus amylovorus	*Streptococcus thermophilus*	*Lactobacillus casei*	*Carnobacterium maltaromaticum*
Lactobacillus rhamnosus	*Clostridium perfringens*	*Streptococcus pneumoniae*	*Clostridium botulinum*
Streptococcus pneumoniae TIGR4	*Lactobacillus reuteri*	*Clostridium tyrobutyricum*	*Clostridium beijerinckii*

Table S.2: Collection of 18 peptides from the database with a described role in alleviating Parkinson's Disease pathology.

aSynPEP ID	Peptide name	DOI publication	Authors statement
ASYNP001	Neuropeptide Y	10.3389/fnagi.2021.646726	"Neuropeptide Y has been demonstrated to exert its potent neuroprotective effects via a variety of pathways related to PD. Decressac et al. (2012) first reported that NPY exerted its neuroprotective effects in both in vitro and in vivo 6-OHDA-induced models of PD."
ASYNP005	Urocortin-2	10.1038/sj.npp.1301123	"Urocortin 2, a new member of the corticotrophin-releasing factor (CRF) neuropeptide family, was reported to be widely expressed in the central nervous system and peripheral tissues. [...] Furthermore, we observed its effects on intracellular Ca(2+) concentration ([Ca(2+)](i)) using confocal microscopy and flow cytometry and on voltage-gated calcium channel (VGCC) currents using whole-cell patch clamp. [...] As calcium overload play a key role in some neuronal degenerative diseases such as Alzheimer's and Parkinson's diseases, our results suggest that urocortin 2 may be a potentially interesting agent for the treatment of these diseases."
ASYNP006	Urocortin	10.1111/j.1460-9568.2007.05653.x	"The potential neuroprotective action of the corticotrophin-releasing factor-related peptide urocortin (UCN) was investigated in the rat 6-hydroxydopamine (6-OHDA) and lipopolysaccharide (LPS) paradigms of Parkinson's disease. [...] Critically, UCN was effective in reversing lesion-induced deficits when given either at the same time as or 7 days after the neurotoxic insult. [...] The apparent ability of UCN to arrest the progression of or even reverse nigral lesions once established suggests that pharmacological manipulation of this system could have substantial therapeutic utility."
ASYNP012	Orexin-B	10.1152/ajpcell.00125.2019	"Orexin is a peptide neurotransmitter released in the globus pallidus. Morphological evidence reveals that both orexin 1 receptor (OX1R) and orexin 2 receptor (OX2R) exist in the globus pallidus. Here we showed that bilateral microinjection of both orexin-A and orexin-B into the globus pallidus alleviated motor deficits in 1-methyl-4-phenyl-1,2,3,6-tetrahydropyridine (MPTP)-induced parkinsonian mice."
ASYNP013	Orexin-A	10.1152/ajpcell.00125.2019	"Orexin is a peptide neurotransmitter released in the globus pallidus. Morphological evidence reveals that both orexin 1 receptor (OX1R) and orexin 2 receptor (OX2R) exist in the globus pallidus. Here we showed that bilateral microinjection of both orexin-A and orexin-B into the globus pallidus alleviated motor deficits in 1-methyl-4-phenyl-1,2,3,6-tetrahydropyridine (MPTP)-induced parkinsonian mice."

ASYNP014	Peptide YY	10.1538/expanim.19-0153	“The non-motor symptoms (NMS) of Parkinson’s disease (PD) are found in more than 90% of patients with PD. Here, we explored the effects of electroacupuncture (EA) stimulation at Zhong wan (CV-12), Qihai (RN-7),Zusanli (ST-36) and Taichong (LR-3) on NMS and brain-gut peptides of PD. [...] EA treatment contributed to alleviating PD by regulating brain-gut peptides in rats, such as NPY, CCK, SST, GAS, and PYY. In conclusion, EA stimulation at CV-12, RN-7, ST-36, and LR-3 effectively alleviates the NMS of PD partly through regulating the levels of brain-gut peptides.”
ASYNP018	Melanin-concentrating hormone	10.1007/s12035-016-0258-8	“Acupuncture has shown the therapeutic effect on various neurodegenerative disorders including Parkinson’s disease (PD). While investigating the neuroprotective mechanism of acupuncture, we firstly found the novel function of melanin-concentrating hormone (MCH) as a potent neuroprotective candidate. [...] A novel finding is that MCH showed a beneficial role in dopaminergic neuron protection via downstream pathways related to neuronal survival.”
ASYNP021	Vasoactive intestinal peptide (VIP)	10.2174/187152710793361595	“Vasoactive intestinal peptide (VIP) is a basic 28 amino acid peptide that binds to a member of the class II family of G protein-coupled receptors (GPCRs). [...] As VIP has been shown to protect against neuronal cell death and act as a modulator of the inflammatory immune response, it has been implicated as a viable treatment option for Parkinson’s disease. In a 2003 study, Delgado and Ganea demonstrated that VIP could protect dopaminergic cells from bacterial endotoxin LPS-induced inflammation in mouse embryonic neurons. [...] In a study using a rat model of Parkinson’s disease, systemically administered VIP was successful at reversing motor deficits but did not stop the decline in striatal dopamine levels. In addition, the peptide was found to preserve neurons by possibly inducing the secretion of neuroprotective agents from brain mast cells, such as nerve growth factor (NGF). In a similar study using a mouse model of PD, VIP treatment significantly decreased dopaminergic neuronal loss in the substantia nigra.”

ASYNP024	Secretin	10.1385/nmm:7:1-2:003	"G protein-coupled receptors (GPCRs) play pivotal roles in regulating the function and plasticity of neuronal circuits in the nervous system. Among the myriad of GPCRs expressed in neural cells, class II GPCRs which couples predominantly to the Gs-adenylate cyclase-cAMP signaling pathway, have recently received considerable attention for their involvement in regulating neuronal survival. [...] Neuropeptides that activate class II GPCRs include secretin, glucagon-like peptides (GLP-1 and GLP-2), growth hormone-releasing hormone (GHRH), pituitary adenylate cyclase activating peptide (PACAP), corticotropin-releasing hormone (CRH), vasoactive intestinal peptide (VIP), parathyroid hormone (PTH), and calcitonin-related peptides. [...] Many of the peptides that activate class II GPCRs promote neuron survival by increasing the resistance of the cells to oxidative, metabolic, and excitotoxic injury."
ASYNP025	Pituitary adenylate cyclase-activating polypeptide 38	10.1124/jpet.106.102236	"Microglial activation is implicated in the progressive nature of numerous neurodegenerative diseases, including Parkinson's disease. Using primary rat mesencephalic neuron-glia cultures, we found that pituitary adenylate cyclase-activating polypeptide (PACAP) 38, PACAP27, and its internal peptide, Gly-Ile-Phe (GIF; PACAP4-6), are neuroprotective at 10(-13) M against lipopolysaccharide (LPS)-induced dopaminergic (DA) neurotoxicity, as determined by [(3)H]DA uptake and the number of tyrosine hydroxylase-immunoreactive neurons."
ASYNP026	Pituitary adenylate cyclase-activating polypeptide 27	10.1124/jpet.106.102236	"Microglial activation is implicated in the progressive nature of numerous neurodegenerative diseases, including Parkinson's disease. Using primary rat mesencephalic neuron-glia cultures, we found that pituitary adenylate cyclase-activating polypeptide (PACAP) 38, PACAP27, and its internal peptide, Gly-Ile-Phe (GIF; PACAP4-6), are neuroprotective at 10(-13) M against lipopolysaccharide (LPS)-induced dopaminergic (DA) neurotoxicity, as determined by [(3)H]DA uptake and the number of tyrosine hydroxylase-immunoreactive neurons."
ASYNP028	Oxyntomodulin (By similarity)	10.1016/j.ejphar.2015.08.038	"Oxyntomodulin is a hormone and a growth factor. It activates two receptors, the Glucagon-like peptide 1 (GLP-1) and the glucagon receptor. [...] D-Ser2-oxyntomodulin (Oxy) is a protease resistant oxyntomodulin analogue that has been developed to treat diabetes. Here we demonstrate for the first time that such analogues have neuroprotective effects. The drug showed protective effects in the 1-methyl-4-phenyl-1,2,3,6-tetrahydropyridine (MPTP) mouse model of PD. [...] The results demonstrate that oxyntomodulin analogues show promise as a novel treatment of PD."

ASYNP029	Glucagon-like peptide 1	10.1016/j.arr.2023.101979	“Therapeutic strategies for neurodegenerative disorders have commonly targeted individual aspects of the disease pathogenesis to little success. Neurodegenerative diseases, including Alzheimer’s disease (AD) and Parkinson’s disease (PD), are characterized by several pathological features. […] Targeting cerebral insulin signalling produces numerous neuroprotective effects in preclinical AD/PD brain models. Clinical trials have shown the promise of approved diabetic compounds in improving motor symptoms of PD and preventing neurodegenerative decline, with numerous further phase II trials and phase III trials underway in AD and PD populations. Alongside insulin signalling, targeting incretin receptors in the brain represents one of the most promising strategies for repurposing currently available agents for the treatment of AD/PD. Most notably, glucagon-like-peptide-1 (GLP-1) receptor agonists have displayed impressive clinical potential in preclinical and early clinical studies. […] Whilst in PD, the GLP-1 receptor agonist exenatide is effective in restoring motor function and cognition.”
ASYNP083	Adrenomedullin	10.1016/S1937-6448(08)01001-0	“Neurotrophic factor production is also considered to play a key role in the therapeutic effects of intracerebral carotid body grafts in Parkinson’s disease. Future research should also focus on trophic actions on carotid body type I cells by peptide neuromodulators, which are known to be present in the carotid body and to show trophic effects on other cell populations, that is, angiotensin II, adrenomedullin, bombesin, calcitonin, calcitonin gene-related peptide, cholecystokinin, erythropoietin, galanin, opioids, pituitary adenylate cyclase-activating polypeptide, atrial natriuretic peptide, somatostatin, tachykinins, neuropeptide Y, neurotensin, and vasoactive intestinal peptide.”
ASYNP089	LL-37	10.1016/j.tibs.2022.02.001	“Alpha-Synuclein (a-syn) oligomers and fibrils are behind neurodegeneration in Parkinson’s disease (PD), but therapeutically targeting them is challenging. Amphipathic and cationic helical peptides inhibit amyloid formation and suppress neurotoxicity by selectively binding the solvent-accessible regions in these toxic species. Can endogenous peptides, like LL-37, constitute a new therapeutic paradigm in PD?”
ASYNP113	Anticancer peptide; LL37	10.1016/j.tibs.2022.02.001	“Alpha-Synuclein (a-syn) oligomers and fibrils are behind neurodegeneration in Parkinson’s disease (PD), but therapeutically targeting them is challenging. Amphipathic and cationic helical peptides inhibit amyloid formation and suppress neurotoxicity by selectively binding the solvent-accessible regions in these toxic species. Can endogenous peptides, like LL-37, constitute a new therapeutic paradigm in PD?”

ASYNP122	Blood-brain barrier peptide; Neuropeptide Y	10.3389/fnagi.2021.646726	“Neuropeptide Y has been demonstrated to exert its potent neuroprotective effects via a variety of pathways related to PD. Decressac et al. (2012) first reported that NPY exerted its neuroprotective effects in both in vitro and in vivo 6-OHDA-induced models of PD.”
ASYNP123	Blood-brain barrier peptide; Exendin-4	10.2174/1566524023666230529093314	“To date, there are no effective therapies with underlying shreds of evidence that alters the progression of PD. Exendin-4, a glucagon-like peptide 1 (GLP-1) receptor agonist, has gained attention for its tremendous neuroprotective potential against numerous neurodegenerative disorders, including PD.”
